# PACSIN2 regulates mercaptopurine cytotoxicity and cellular biomechanics through Rac1 modulation in intestinal epithelial cells

**DOI:** 10.3389/fphar.2026.1860702

**Published:** 2026-09-01

**Authors:** Alessia Norbedo, Giulia Zudeh, Donatella Fiducioso, Beatrice Senigagliesi, Pietro Parisse, Loredana Casalis, Giuliana Decorti, Marianna Lucafò, Gabriele Stocco

**Affiliations:** 1 Department of Medicine Surgery and Health Sciences, University of Trieste, Trieste, Italy; 2 Department of Translational and Advanced Diagnostics, Institute for Maternal and Child Health I.R.C.C.S. Burlo Garofolo, Trieste, Italy; 3 Department of Life Sciences, University of Trieste, Trieste, Italy; 4 Joint Research Center, Ispra, Italy; 5 Istituto Officina dei Materiali, Consiglio Nazionale delle Ricerche (CNR - IOM), Trieste, Italy; 6 Elettra Sincrotrone Trieste, Trieste, Italy

**Keywords:** biomechanical properties, inflammatory bowel disease, PACSIN2, Rac1, thiopurines

## Abstract

**Introduction:**

Mercaptopurine (MP) is used to treat pediatric acute lymphoblastic leukemia and inflammatory bowel disease (IBD). The Protein Kinase C and Casein Kinase Substrate in Neurons 2 (PACSIN2) rs2413739 polymorphism is an expression-quantitative trait locus associated with reduced PACSIN2 expression in gastrointestinal tissues and thiopurine-related gastrointestinal toxicity. PACSIN2 interacts with Rac1, a key thiopurine target protein involved in cytoskeletal regulation.

**Methods:**

LS180 intestinal cells with stable PACSIN2 knockdown (KD) or transient overexpression were used to assess the role of PACSIN2 in regulating Rac1 under basal conditions and after MP exposure. Rac1 levels and activity were measured by Western blot and G-LISA assay, respectively. Cytotoxicity was evaluated by MTT assay following exposure to MP and the Rac1 inhibitor NSC23766. Cell stiffness was assessed by atomic force microscopy, and cytoskeletal organization was examined by immunofluorescence.

**Results:**

PACSIN2 KD cells exhibited elevated Rac1 expression and activity, and increased sensitivity to MP, which was further augmented by the Rac1 inhibitor NSC23766. Conversely, PACSIN2 overexpression reduced Rac1 levels and conferred MP resistance. PACSIN2 depletion decreased cell stiffness affecting the cytoskeletal architecture, particularly impairing tubulin levels and actin distribution. MP exposure reduced cell stiffness and decreased both Rac1 expression and activity across all tested cell lines, with more pronounced effects in LS180 KD cells. Altered cytoskeletal organization, especially actin redistribution, correlated with changes in cellular biomechanical properties and drug sensitivity.

**Conclusion:**

These findings suggest that PACSIN2 modulates MP response by regulating Rac1 levels and cytoskeletal integrity, providing a molecular basis for the clinical association between PACSIN2 and thiopurine-related gastrointestinal toxicity. These insights may contribute to the development of personalized approaches to optimize thiopurine therapy in pediatric patients.

## 1 Introduction

Protein kinase C and casein kinase substrate in neuron 2 (PACSIN2), also known as syndapin II, is an F-BAR protein involved in cytoskeletal organization, vesicle trafficking, endocytosis, and cell migration (Quan and Robinson, 2013), as well as in autophagy (Zudeh et al., 2023).

PACSINs comprise three paralogs, all characterized by an N-terminal F-BAR-domain, a C-terminal Src-homology3-domain (SH3) domain, and a variable central region containing asparagine-proline-phenylalanine (NPF) motifs in PACSIN1 and PACSIN2 (Dumont and Lehtonen, 2022). The F-BAR domain mediates membrane curvature, tubulation, and invagination during vesicle budding, endocytosis, and caveolae biogenesis, and it is responsible for the interaction between PACSIN2 and filamin A (Begonja et al., 2015). The SH3-domain, in turn, regulates PACSIN2 autoinhibition and mediates the interactions with proteins such as dynamin and Rac1 (Quan and Robinson, 2013). Specifically, the interaction between the SH3 domain of PACSIN2 and the C-terminal domain of Rac1 directly modulates Rac1 activity (de Kreuk et al., 2011). Additionally, PACSIN2 may affect Rac1 indirectly, through dynamin regulation (Razidlo et al., 2013). PACSIN2 is ubiquitously expressed in eukaryotes and localizes to plasma and endosomal membranes, where it interacts with Rac1 in intracellular tubulin structures and early endosomes (Razidlo et al., 2013). Through modulation of Rac1 expression and activity, PACSIN2 influences cell migration, morphology, biomechanical properties, endothelial adhesion (Wint et al., 2023), and cell spreading (Razidlo et al., 2013). Rac1 is a small GTPase of the Rho family that controls actin cytoskeleton dynamics and regulates cell spreading and migration (Nguyen et al., 2016). Cellular mechanical properties are linked to different biological functions, including cellular morphology (Postema et al., 2019), differentiation (He et al., 2023), motility (Sens and Plastino, 2015), proliferation (Mahajan et al., 2019), and mechanotransduction (Sotodosos-Alonso et al., 2023). Cell deformation is predominantly governed by actin filaments, whose organization depends on actin concentration, cross-linking density, and mechanical stress (Postema et al., 2019). PACSIN2 plays an essential role in intestinal homeostasis, being predominantly expressed in enterocyte microvilli, where it co-localizes with F-actin (Dumont and Lehtonen, 2022). PACSIN2 knockout mice show reduced microvillar density and loss of the typical hexagonal microvillar organization (Postema et al., 2019). Disruption of normal microvillar architecture compromises the intestinal barrier, contributing to the onset of pathological conditions, such as inflammatory bowel diseases (IBD) (Dumont and Lehtonen, 2022; VanDussen et al., 2018).

Among the techniques developed to study cellular biomechanical properties, atomic force microscopy (AFM) is an established high-resolution method based on measuring the sample response to the force exerted by an indenting probe (Mierke, 2020). Contact-model analyses of indentation measurements allow quantification of cellular plasticity, elasticity, and adhesion properties (Mierke, 2020), which may change in response to Rac1 expression and activity (Kunschmann et al., 2019).

Thioguanine (TG), mercaptopurine (MP), and azathioprine (AZA) are thiopurine drugs with immunosuppressive properties. MP is used in the treatment of acute lymphoblastic leukemia (ALL), whereas AZA is administered to maintain remission in patients with IBD (Franca et al., 2019). Their active metabolites, the thioguanine nucleotides (TGN), exert cytotoxic effects through DNA incorporation, inhibition of the *de novo* purine synthesis, and inhibition of Rac1, the latter inducing apoptosis in intestinal LS180 cells (Zudeh et al., 2023).

Thiopurines metabolism involves several enzymes whose activity displays inter-individual variability on a genetic basis, contributing to suboptimal treatment responses in approximately 40%–60% of patients (González-Lama and Gisbert, 2016; van Hoeve and Vermeire, 2020). The PACSIN2 rs2413739 variant has been associated with increased gastrointestinal toxicity in patients with ALL receiving MP (frequency: 2.1%, 9.1% and 13.2% in CC, CT and TT carriers, respectively) and with reduced AZA efficacy in IBD patients (clinically active disease in 18.2%, 28.4%, and 43.1% of CC, CT, and TT patients, respectively) (Franca et al., 2020). This variant is an expression quantitative trait locus (eQTL) associated with reduced PACSIN2 expression in gastrointestinal tissues, including oesophagus mucosa (NES = −0.069, P = 0.0092) (GTEx Consortium, 2013). Notably, PACSIN2 knockdown (KD) increased MP cytotoxicity in intestinal LS180 cells but not in lymphoid cells, suggesting a tissue-specific role of PACSIN2 in the intestinal response to thiopurines (Zudeh et al., 2023). Based on these findings, it has been hypothesized that PACSIN2 influences thiopurine cytotoxicity by modulating Rac1 activity (Sotodosos-Alonso et al., 2023), which may in turn affect the biomechanical properties of intestinal epithelial cells (Martínez-Sánchez et al., 2023). However, the precise mechanisms linking PACSIN2 expression, cytoskeletal integrity, and thiopurine sensitivity in the intestinal epithelium remain unclear.

This study aimed to evaluate *in vitro* the impact of PACSIN2 modulation (KD and overexpression) on Rac1 expression and activity, cellular stiffness and MP cytotoxicity in LS180 cells, with the goal of clarifying the molecular mechanisms underlying inter-individual variability in thiopurine response.

## 2 Materials and methods

### 2.1 Cell culture

Human intestinal LS180 cells were transfected with lentivirus encoding shRNA (GAA​CGA​TGA​CTT​CGA​GAA​GAT) to determine the stable *PACSIN2* KD (KD cells) or a lentivirus encoding control scramble shRNA (MOCK cells) at St. Jude Children’s Research Hospital (SJCRH) of Memphis and were kindly provided by Prof. W. E. Evans (SJCRH, Memphis, Tennessee, United States) (Zudeh et al., 2023). The cells were mycoplasma-negative. Both cell lines were cultured in RPMI 1640 (*EuroClone, Milan, Italy*) with the addition of 10% fetal bovine serum (FBS) (*Merck, Saint Louis, MO,* United States), 1% of L-glutamine 200 mM (*EuroClone, Milan, Italy*), 1% of penicillin 10,000 UI/mL and streptomycin 10,000 μg/mL (*EuroClone, Milan, Italy*) at 37 °C with 5% CO_2_. Puromycin (0.005 mg/mL) (*Merck, Saint Louis, MO,* United States) was added to the complete culture medium in order to select engineered LS180 cell lines.

### 2.2 Transient transfection

LS180 MOCK cells were cultured in RPMI 1640 without antibiotics and seeded at 1 × 10^6^ cells/well to be transfected with 7 μg of the OmicsLink Expression-Ready ORF cDNA Vector encoding for the PACSIN2 wild-type cDNA (Cat# CS-I2075-M39, *GeneCopoeia, Rockville, MD,* United States) and the respective scramble vector (Cat# EX-EGFP-M39, *GeneCopoeia, Rockville, MD,* United States), used as empty control, using Lipofectamine 2000 Transfection Reagent (11668019; *Thermo Fisher Scientific, Waltham, MA,* United States) and Opti-MEM medium (31985070; *Thermo Fisher Scientific, Waltham, MA,* United States). After 48 h cells were collected and seeded for immunoblotting and cytotoxicity assays.

### 2.3 Cytotoxicity assay

Cells were seeded in 96 well plate (5,000 cells/well) and treated for 24, 48, and 72 h with different concentrations of MP (0.3 µM, 0.6 µM, 1.25 µM, 2.5 µM, 5 μM, 10 μM, 20 μM, 40 μM, 80 μM, 160 µM) or the Rac1 inhibitor NSC23766 (0.3 µM, 0.6 µM, 1.25 µM, 2.5 µM, 5 μM, 10 μM, 20 μM, 40 μM, 80 μM, 160 µM). Also the co-treatment with MP 1.25 µM and NSC23766 2.5 µM was tested.

Four hours (h) before the end of the incubation, MTT solution 3-(4,5-dimethylthiazol-2-yl)-2,5-diphenyltetrazolium bromide (*Merck, Saint Louis, MO,* United States) was added at a final concentration of 500 μg/mL. Formazan salts produced by viable cells were solubilized in dimethyl sulfoxide (*Merck, Saint Louis, MO,* United States), and the absorbance at 540–630 nm was detected using a FLUOstar Omega spectrophotometer *(BMG Labtech, Ortenberg*).

### 2.4 Western blot analysis

Cells were collected and suspended in 100 µL *Lysis Buffer* (1 mM EDTA (*Merck, Saint Louis, MO,* United States), 150 mM NaCl (*Merck, Saint Louis, MO,* United States), 50 mM Tris-HCl (*Merck, Saint Louis, MO,* United States), sodium deoxycholate 0.5% (*Merck, Saint Louis, MO,* United States), NP-40 1% (*Merck, Saint Louis, MO,* United States) completed with protease inhibitor cocktail 1X *(Merck, Saint Louis, MO,* United States), and protein lysates were quantified by Bradford assay (*Merck, Saint Louis, MO,* United States).

Ten µg of proteins were separated on Sodium Dodecyl Sulphate–Polyacrylamide Gel Electrophoresis (SDS-PAGE) 4%–12% (*Bolt 4%–12% Bis-Tris Plus Gels, Thermo Fisher Scientific, Waltham, MA,* United States) and transblotted to nitrocellulose membranes (*Thermo Fisher Scientific, Waltham, MA,* United States), which were incubated with 5% milk in Tris-buffered saline buffer (0.012% p/V Trizma (*Merck, Saint Louis, MO,* United States); 0.009% p/V NaCl (*Merck, Saint Louis, MO,* United States) completed with 0.001% Tween 20 (*Merck, Saint Louis, MO,* United States). Blots were cut prior to antibody hybridization. Incubation with primary antibodies was performed overnight at 4 °C. The tested antibodies were mouse anti-human Rac1 (1:800 dilution (*Cytoskeleton, Denver, CO,* United States), rabbit anti-human dynamin (1:1,000 dilution, *Thermo Fisher Scientific, Waltham, MA,* United States), rabbit anti-human GAPDH (1:1,000 dilution, *Santa Cruz, Dallas, TX,* United States), rabbit anti-human actin (1:1,000 dilution, *Abcam, Cambridge, UK*), rabbit anti-human PACSIN2 (1:1,000 dilution; *OriGene, MD,* United States). The appropriate secondary antibody was incubated for 1 h at 4 °C: Horse anti-mouse IgG (1:8,000 dilution, *Cell Signaling, Danvers, MA,* United States) or Goat anti-rabbit IgG (1:10000 dilution, *Merck, Saint Louis, MO,* United States). Immunocomplexes were visualized by chemiluminescence using the *LiteABLOT Turbo Chemiluminescent Substrate* (*EuroClone, Milan, Italy*) and *ChemiDoc™ MP Imaging System* (*Bio-Rad Laboratories*).

### 2.5 Immunofluorescence

LS180 MOCK, LS180 KD, LS180 EMPTY, and LS180 PACSIN2 OVEREXPRESSION cells were seeded at 0.2 × 10^6^ cells/well, treated for 48 h with IC20 and IC50 of MP and fixed using 4% paraformaldehyde (PFA) for approximately 45 min at room temperature. Permeabilization was performed using 0.2% Triton-X in PBS for 45 min at room temperature. After three washes with PBS, cells were blocked with a solution formed by 1% bovine serum albumin (BSA) and 0.01% Triton-X in PBS for 1 h at room temperature. After blocking, the primary antibody against human α-tubulin (1:500 in blocking solution; *GeneTex*, *Irvine, California,* United States) was incubated overnight at 4 °C. After three washes with PBS, goat anti-mouse Alexa Fluor 488 (*Thermo Fisher Scientific, Waltham, MA,* United States) secondary antibody was incubated for 1 h at 4 °C (1:1,000 dilution in blocking solution). After three washes, phalloidin (1:200 in water Milliq, *Thermo Fisher Scientific, Waltham, MA,* United States) was added for 2 h at 4 °C. After three washes with PBS, DAPI (2-(4-amidinophenyl)-1H-indole-6-carboxamidine) (1:1,000 in H_2_O Milliq *Merck, Saint Louis, MO,* United States) was added for 15 min at 4 °C.

The glass slides were washed three times with PBS and mounted using ProLong™ Gold Antifade reagent (*Thermo Fisher Scientific, Waltham, MA,* United States).

### 2.6 Equipment and setting

For immunoblotting analysis, the blots were acquired using the *ChemiDoc™ MP Imaging System (Bio‐Rad Laboratories)* using the auto‐optimal high‐resolution acquisition setting, which corresponded to 20 s for the housekeeping bands and 100 s for Rac1, PACSIN2 and dynamin bands. All membranes were analysed using *ImageJ®software*.

For immunofluorescence, the specimens were visualized using an Inverted Research Microscope Eclipse Ti (*Nikon*) equipped with an epifluorescence illuminator or a 488 nm laser for Total Internal Reflection Fluorescence application. Optical magnification was 20X or 50X for DAPI and phalloidin (generating images with 0.33 and 0.13 μm/pixel, respectively), whereas tubulin was detected only at ×20 magnification (generating images with 0.33 μm/pixel).

### 2.7 Sample preparation for G-LISA assay

Both LS180 MOCK and KD cells were seeded (1 × 10^6^ cells/well) and treated for 24, 48 and 72 h with MP at three different concentrations IC20, IC50 and IC80, obtained from the MTT assay results (2.04 μM, 3.23 μM, 5.66 μM for LS180 MOCK cells and 1.28 μM, 2.18 μM, 3.70 μM for LS180 KD cells, respectively). Samples were lysed and quantified using *Precision Red^TM^ Advanced Protein Assay Reagent* (*Cytoskeleton, Denver, CO,* United States), and the absorbance at 490 nm was quantified using a *FLUOstar Omega* spectrophotometer *(BMG Labtech, Ortenberg)*. The same protocol was used for LS180 MOCK cells transfected with the plasmid encoding PACSIN2 wild-type or the empty plasmid for 48 h after transfection and after an additional 72 h, which corresponded to the time point used for the cytotoxicity assay.

### 2.8 G-LISA assay

The Rac1 G-LISA® kit (*Cytoskeleton, Denver, CO,* United States) was used to specifically detect active GTP-bound Rac1 and distinguish it from Rac2 and Rac3 isoforms in incubated cell/tissue lysates at a concentration of 1 mg/mL. The amount of active Rac1 was detected using the mouse anti human-Rac1 antibody (*ARC03, Cytoskeleton, Denver, CO,* United States), according to the manufacturer’s instructions.

### 2.9 Cell morphology analysis after drug exposure

LS180 MOCK and LS180 KD cells were seeded at 1 × 10^6^ cells/well and exposed to the respective IC20, IC50, and IC80 concentrations of MP for 24, 48, and 72 h. At each time point, cell morphology was observed using the EVOS M5000 Imaging System (*Thermo Fisher Scientific, Waltham, MA,* United States) at ×40 magnification.

### 2.10 Biomechanical analysis

AFM was used to evaluate cell line stiffness (described as Young’s modulus or elastic modulus) (Ding et al., 2017) through force spectroscopy analysis, which was carried out using the Smena AFM (NT-MDT Co., Moscow, Russia) mounted on an inverted fluorescence microscope (Nikon Eclipse Ti-U).

For the sample preparation, LS180 MOCK and KD cell lines were seeded at 1.5 × 10^6^ cells/glass and fixed with PFA 4% for 20 min by shaking at room temperature. To visualize the nuclei, cells were stained with DAPI (*Merck, Saint Louis, MO,* United States). For AFM force spectroscopy, silicon spherical tips with a diameter of 20 µm (Tip: CSG01 cantilever from NT-MDT Smena, spring constant k = 0.006–0.007 N/m) were used to obtain the global elastic modulus for each single cell. To determine cell stiffness, in correspondence of cell nucleus a force of 0.7 nN, an indentation rate of 2 μm/s and an indentation of −500 nm were applied and the elastic modulus values (E), in KPa, were determined by fitting the obtained force-displacement curves with a Hertz model (Ladjal et al., 2009) by using *AtomicJ® software.* Three independent biological replicates were used for each experiment. For each biological replicate, three slides were prepared per experimental condition (technical triplicates), and 100 cells were analyzed per slide. Thus, 300 cells were quantified for each condition in each biological replicate. Data represent the mean of three biological replicates, corresponding to a total of 900 cells analyzed per condition.

### 2.11 Statistical analysis

Statistical analyses (t-test, one-way ANOVA with Tukey post-test, and two-way ANOVA with Tukey post-test) were performed using GraphPad Prism version 8.0 and RStudio version 4.2.2. Data are reported as mean ± standard deviation (SD). Statistical significance was set at P < 0.05.

## 3 Results

### 3.1 Cell line characterization

A significant reduction of PACSIN2 in LS180 KD cells confirmed PACSIN2 stable silencing (P < 0.0001, t-test Supplementary Figure S1A), whereas 48 h after cell transfection with a plasmid encoding PACSIN2 (LS180 PACSIN2 OVEREXPRESSION cells) or an empty control (LS180 EMPTY cells) confirmed PACSIN2 overexpression (P = 0.03, t-test Supplementary Figure S1B).

### 3.2 The MP exposure effects on PACSIN2 levels

**FIGURE 1:**
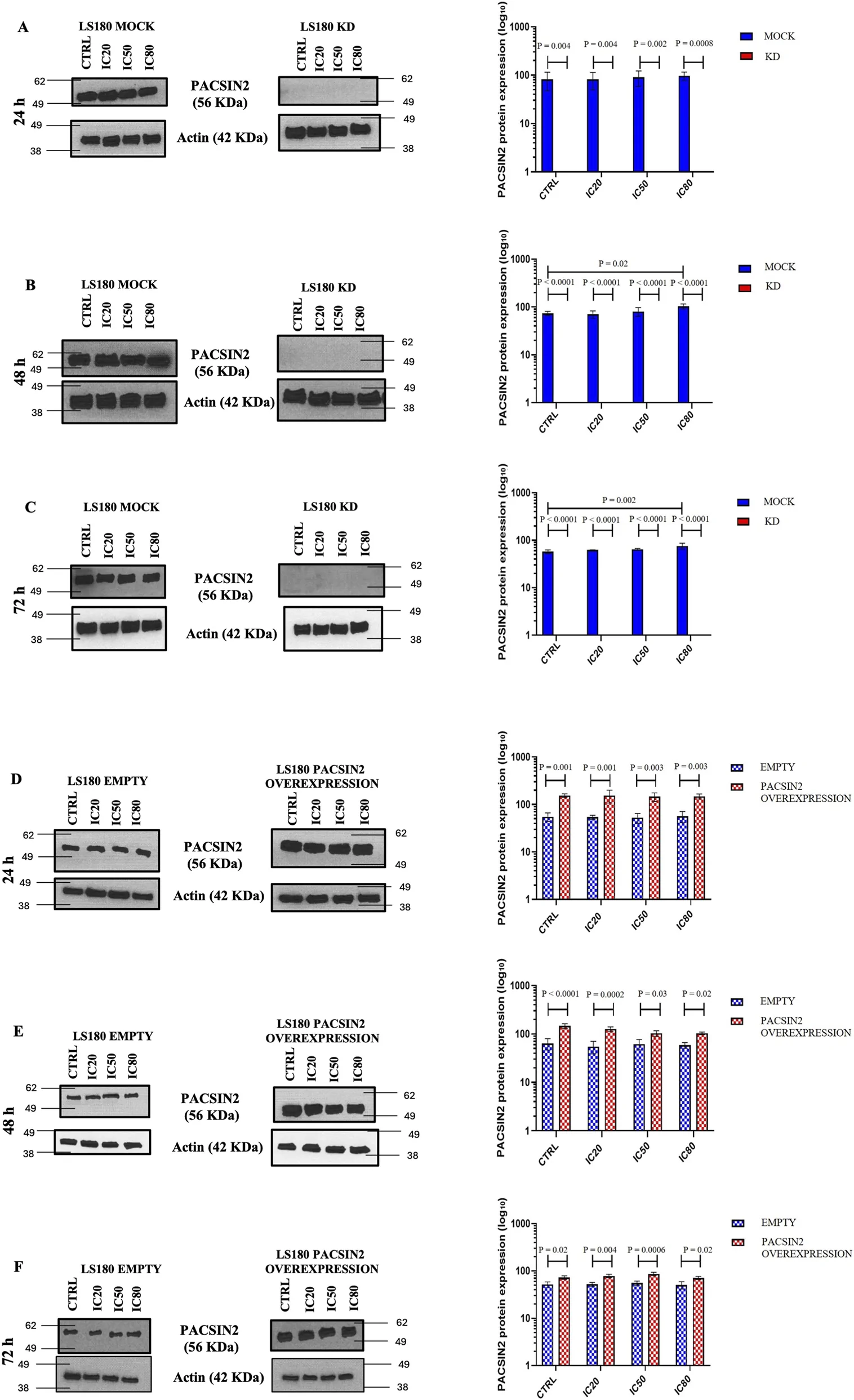
The effect of mercaptopurine (MP) on PACSIN2 levels. **(A)** Immunoblotting results of PACSIN2 protein levels in LS180 MOCK, LS180 KD treated with MP at IC20, IC50, IC80 for 24 h. Data were normalized against β-actin (data expressed as mean ± standard deviation, n = 3, t-test). **(B)** Immunoblotting results of PACSIN2 protein levels in LS180 MOCK, LS180 KD treated with MP at IC20, IC50, IC80 for 48 h. Data were normalized against β-actin (data expressed as mean ± standard deviation, n = 3, t-test). **(C)** Immunoblotting results of PACSIN2 protein levels in LS180 MOCK, LS180 KD treated with MP at IC20, IC50, IC80 for 72 h. Data were normalized against β-actin (data expressed as mean ± standard deviation, n = 3, t-test). **(D)** Immunoblotting results of PACSIN2 protein levels in LS180 EMPTY and LS180 PACSIN2 OVEREXPRESSION cells treated with MP at IC20, IC50, IC80 for 24 h. Data were normalized against β-actin (data expressed as mean ± standard deviation, n = 3, t-test). **(E)** Immunoblotting results of PACSIN2 protein levels in LS180 EMPTY and LS180 PACSIN2 OVEREXPRESSION cells treated with MP at IC20, IC50, IC80 for 48 h. Data were normalized against β-actin (data expressed as mean ± standard deviation, n = 3, t-test). **(F)** Immunoblotting results of PACSIN2 protein levels in LS180 EMPTY and LS180 PACSIN2 OVEREXPRESSION cells treated with MP at IC20, IC50, IC80 for 72 h. Data were normalized against β-actin (data expressed as mean ± standard deviation, n = 3, t-test).

PACSIN2 levels were evaluated in LS180 MOCK, KD, EMPTY, and PACSIN2 OVEREXPRESSION cells after MP exposure at IC20, IC50, and IC80 for 24, 48, and 72 h. Drug exposure did not significantly alter PACSIN2 levels (Figure 1).

### 3.3 PACSIN2 effects on cytotoxicity of MP and the Rac1 inhibitor NSC23766 in LS180 cells

**FIGURE 2:**
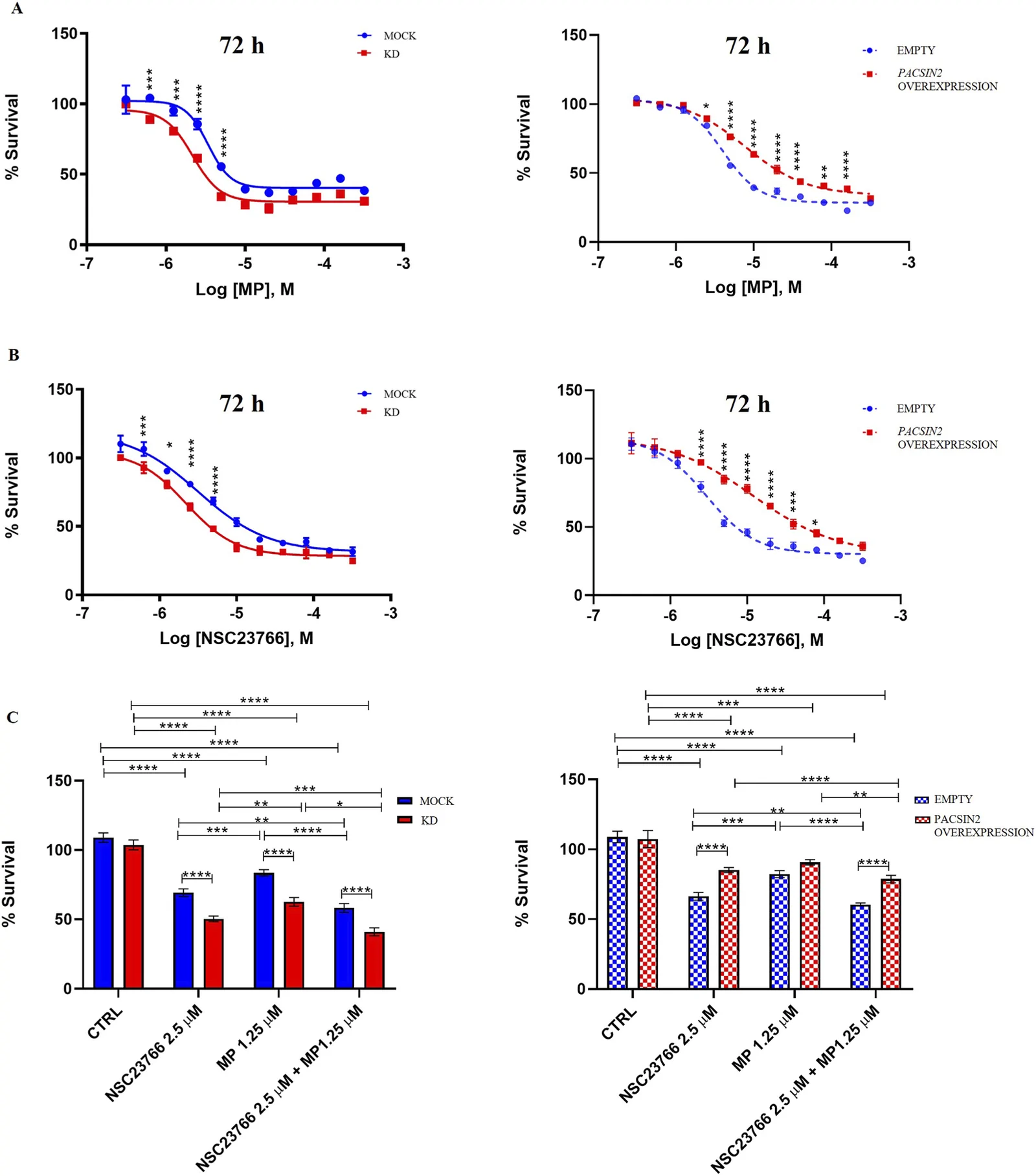
The effect of PACSIN2 on mercaptopurine (MP) and the Rac1 inhibitor NSC23766 cytotoxicity. **(A)** Cytotoxicity MTT dose-response curve of LS180 MOCK, LS180 PACSIN2 KD cells, LS180 MOCK EMPTY, and LS180 MOCK PACSIN2 OVEREXPRESSION cells treated with MP (0.3 µM 160 µM) for 72 h. A significant difference of MP sensitivity between LS180 MOCK and LS180 PACSIN2 KD cells was detected at 0.3 µM (P = 0.001), 0.6 µM (P = 0.001), 1.25 µM (P = 0.0001), 2.5 µM (P = 0.0001). A significant difference of MP sensitivity between LS180 MOCK EMPTY and LS180 MOCK PACSIN2 cells was found at 1.25 µM (P < 0.05), 2.5 µM (P = 0.001), 5 µM (P = 0.0001), 10 µM (P = 0.001) and 20 µM (P = 0.01). Data are expressed as mean ± standard deviation from three independent experiments (Two-way ANOVA, post-test Tukey). **(B)** Cytotoxicity MTT dose-response curves of LS180 MOCK, LS180 PACSIN2 KD cells, LS180 MOCK EMPTY, and LS180 MOCK PACSIN2 OVEREXPRESSION cells treated with the Rac1 inhibitor NSC23766 (0.3 µM 160 µM) for 72 h. A significant difference from the Rac1 inhibitor NSC23766 was found between LS180 MOCK and KD cells (P < 0.0001)) and between LS180 EMPTY cells and LS180 PACSIN2 OVEREXPRESSION cells (P < 0.0001). Data are expressed as mean ± standard deviation from three independent experiments (Two-way ANOVA, post-test Tukey). **(C)** Cytotoxicity MTT dose-response curve LS180 MOCK, LS180 PACSIN2 KD cells, LS180 MOCK EMPTY and LS180 MOCK PACSIN2 OVEREXPRESSION cells co-treated with MP (from 0.3 µM to 160 µM) and the Rac1 inhibitor NSC23766 2.5 µM for 72 h. There was a significant difference between LS180 MOCK and LS180 KD cell lines (P < 0.0001)) and between LS180 MOCK EMPTY and LS180 MOCK PACSIN2 OVEREXPRESSION cell lines (P < 0.0001). Data are expressed as mean ± standard deviation from three independent experiments (Two-way ANOVA, post-test Tukey).

Treatment with MP exerted cytotoxicity over time. After 24 and 48 h of drug exposure the treatment did not reduce cell viability below 50% of survival rate (Supplementary Figure S4A), whereas after 72 h of MP exposure a greater cytotoxic effect was observed (Figure 2A). LS180 KD cells were significantly more sensitive to MP at the 0.3 µM, 0.6 µM, 1.25 µM and 2.5 µM concentrations compared to LS180 MOCK cells (P < 0.001, P < 0.001, P < 0.0001 and P < 0.0001, respectively, two-way ANOVA, post-test Tukey Figure 2A), resulting in distinct IC20, IC50, IC80 values for LS180 MOCK and KD cells (Table SI). Interestingly, PACSIN2 OVEREXPRESSION cells were significantly more resistant to 72 h of MP exposure at 1.25, 2.5, 5, 10, and 20 µM concentrations than LS180 MOCK cells transfected with an empty plasmid used as a control (P < 0.05, P < 0.001, P < 0.0001, P < 0.001, and P < 0.01, respectively; two-way ANOVA, post-test Tukey Figure 2A), showing different IC20, IC50, and IC80 levels (Table SI).

Similar to MP, treatment with the Rac1 inhibitor NSC23766 increased cellular cytotoxicity in LS180 KD compared to LS180 MOCK cells after 72 h of drug exposure (P < 0.0001, Figures 2B,C), whereas LS180 PACSIN2 OVEREXPRESSION cells were more resistant than LS180 EMPTY cells to this compound (P < 0.0001, Figures 2B,C).

An additive effect was shown after co-treatment with MP and the Rac1 inhibitor NSC23766 in LS180 MOCK cells (P < 0.0001, Figure 2C), LS180 KD cells (P < 0.0001, Figure 2C), LS180 EMPTY cells (P < 0.0001, Figure 2C), and LS180 PACSIN2 OVEREXPRESSION cells (P = 0.006, Figure 2C). Interestingly, after co-treatment with MP and the Rac1 inhibitor NSC23766, LS180 KD cells remained more sensitive than LS180 MOCK cells (P < 0.0001, Figure 2C), and LS180 PACSIN2 OVEREXPRESSION cells were more resistant than LS180 EMPTY cells (P < 0.0001, Figure 2C).

### 3.4 PACSIN2 effects on Rac1 protein expression and activity in LS180 cells

**FIGURE 3:**
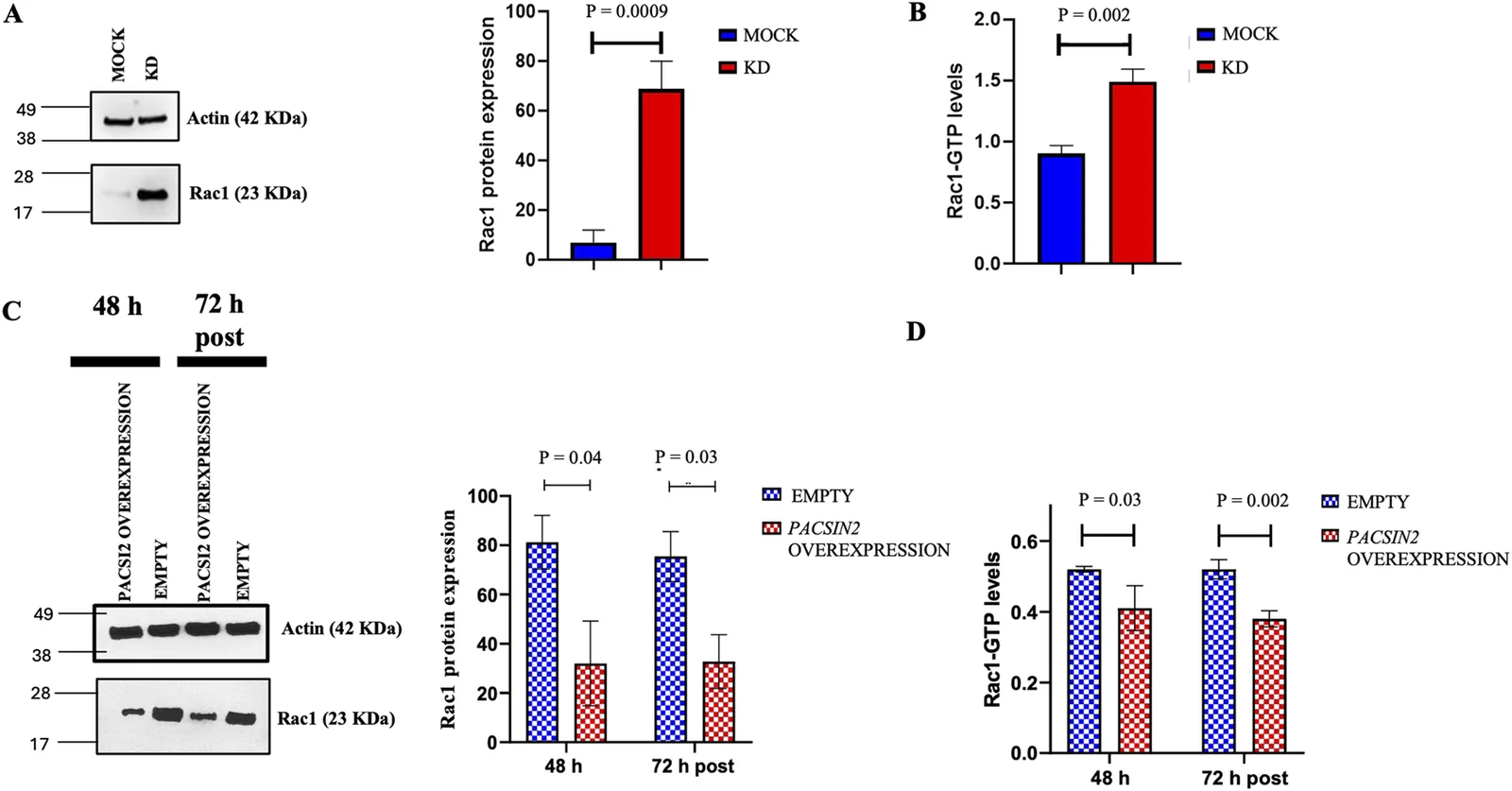
The effect of PACSIN2 on Rac1 expression and activity levels. **(A)** Immunoblotting results of Rac1 protein levels in LS180 MOCK and LS180 PACSIN2 KD cells normalized against β‐actin (data expressed as mean ± standard deviation, n = 3, t‐test). **(B)** Rac1 protein activity evaluated as Rac1‐GTP amount in LS180 MOCK and LS180 PACSIN2 KD cell lines (data expressed as mean ± standard deviation, n = 3, t‐test). **(C)** immunoblotting results of Rac1 protein levels in LS180 MOCK cells transfected with an empty plasmid (LS180 EMPTY) used as control and LS180 cells presenting the PACSIN2 overexpression (LS180 PACSIN2 OVEREXPRESSION) cells at 48 after transfection (48 h) and after additional 72 h (72h post) normalized against β‐actin (data expressed as mean ± standard deviation, n = 3, t‐test). **(D)** Rac1 protein activity evaluated as Rac1‐GTP amount in LS180 EMPTY and LS180 PACSIN2 OVEREXPRESSION cells at 48 after transfection (48 h) and after additional 72 h (72h post, data expressed as mean ± standard deviation, n = 3, t‐test).

Since PACSIN2 interacts with Rac1, the levels of both Rac1 protein expression and activity (expressed as Rac1-GTP levels) were evaluated in LS180 MOCK and KD cells. A significantly higher level of Rac1 protein (Figure 3A) and active Rac1-GTP (Figure 3B) was found in LS180 KD cells compared to LS180 MOCK cells (P = 0.0009 and P = 0.002, respectively; two-way ANOVA, post-test Tukey). Moreover, PACSIN2 OVEREXPRESSION cells showed a significant reduction in Rac1 levels at both 48 and 72 h post transfection compared to LS180 EMPTY cells (P = 0.04 and P = 0.03, respectively; two-way ANOVA, post-test Tukey Figure 3C). In addition, Rac1 activity was significantly reduced in PACSIN2 OVEREXPRESSION cells at both time points compared to LS180 EMPTY cells (P = 0.03 and P = 0.002, respectively; two-way ANOVA, post-test Tukey Figure 3D).

### 3.5 PACSIN2 effects on LS180 cells biomechanical properties evaluated by AFM

**FIGURE 4:**
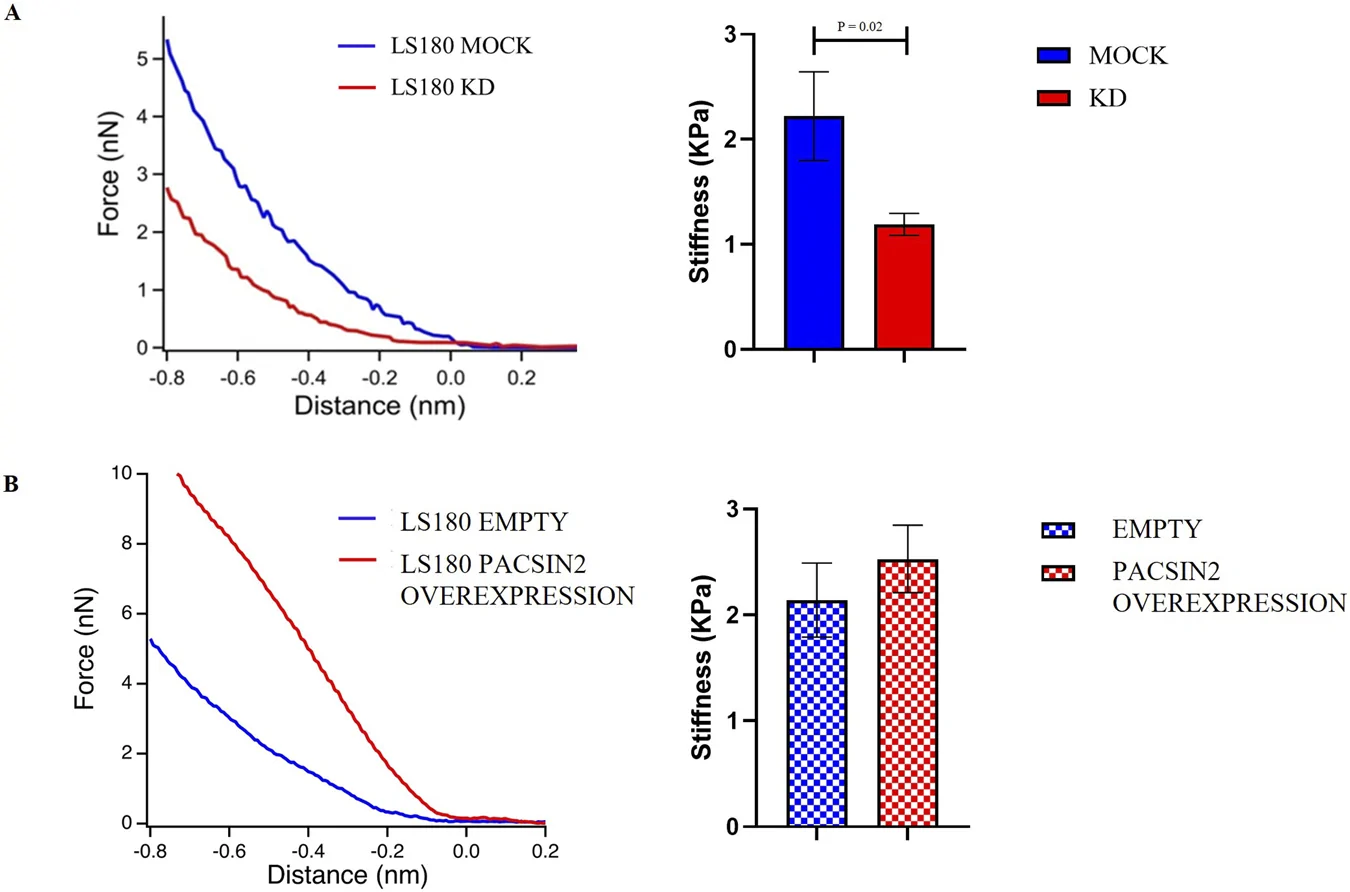
The effect of PACSIN2 on LS180 cells biomechanical properties by atomic force microscopy (AFM). **(A)** Force‐distance curves of LS180 MOCK and LS180 PACSIN2 KD cell lines obtained by AFM to determine the cell stiffness (data expressed as mean ± standard deviation, n = 3, t‐test). **(B)** Force‐distance curves of LS180 EMPTY and LS180 PACSIN2 OVEREXPRESSION cell lines obtained by AFM to determine the cell stiffness (data expressed as mean ± standard deviation, n = 3, t‐test).

Since PACSIN2 interacts with different proteins responsible for actin organization, the effects of PACSIN2 KD and overexpression on the biomechanical properties of LS180 cells were analysed by AFM. The force curve recorded for LS180 MOCK cells was steeper than the one recorded for LS180 KD cells, indicating a lower stiffness in LS180 KD compared to LS180 MOCK cells (1.1 ± 0.04 KPa vs. 2.2 ± 0.03 KPa, P = 0.002, t-test Figure 4A). Consistently, the stiffness was increased in LS180 PACSIN2 OVEREXPRESSION cells compared to LS180 EMPTY cells, although this increase did not reach a significant difference (P = 0.07, t-test Figure 4B).

### 3.6 MP and PACSIN2 effects on Rac1 protein expression and activity levels

**FIGURE 5:**
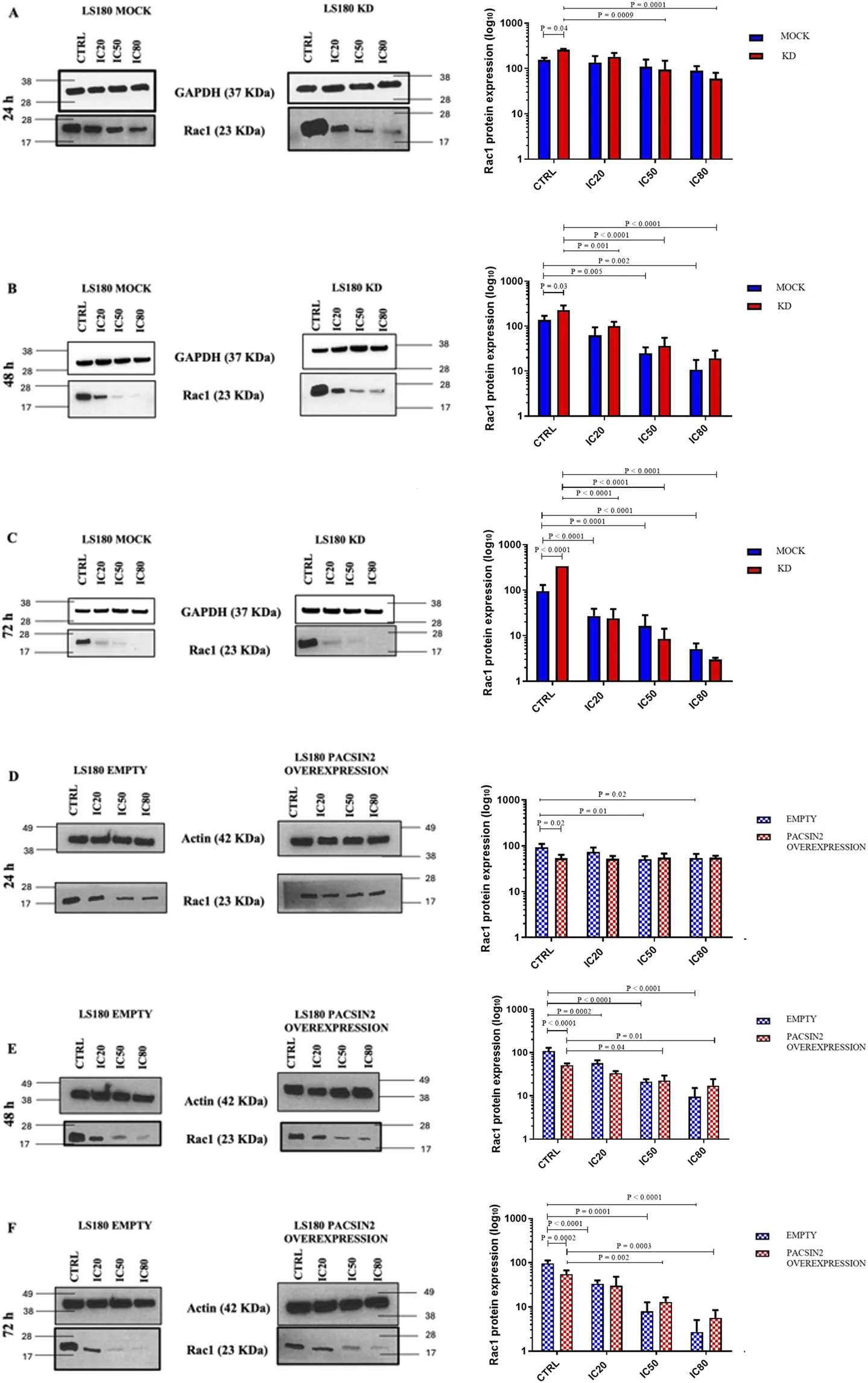
Analysis of Rac1 protein levels under basal condition and after mercaptopurine (MP) treatment. Western Blot analysis of Rac1 protein levels in LS180 MOCK and LS180 PACSIN2 KD cell lines untreated and after 24 h **(A)**, 48 h **(B)** or 72 h **(C)** of MP treatment at the IC20, IC50 and IC80. Western Blot analysis of Rac1 protein levels in LS180 EMPTY and LS180 PACSIN2 OVEREXPRESSION cell lines untreated and after 24 h **(D)**, 48 h **(E)** or 72 h **(F)** of MP treatment at the IC20, IC50 and IC80. Rac1 levels were normalized using GAPDH or β‐actin and expressed in logarithmic scale (data expressed as mean ± standard deviation, n = 3, Two‐way ANOVA, post‐test Tukey). The β‐actin membranes of Panel **(D–F)** are the same presented in Figure 7 Panel **(D–F)**, respectively.

Twenty-four h of MP exposure at IC50 and IC80 significantly reduced Rac1 protein expression in LS180 KD cells, with comparable Rac1 levels in the two cell lines (Figure 5A). After 48 h of MP treatment, a reduction in Rac1 protein expression was observed in both cell lines. In particular, MP at IC50 and IC80 reduced Rac1 in both LS180 MOCK and KD cells, whereas MP IC20 led to lower Rac1 levels in LS180 KD cells only (Figure 5B). After 72 h of treatment, all tested concentration of MP decreased Rac1 protein expression in both cell lines (Figure 5C). Consistently, under basal conditions, LS180 PACSIN2 OVEREXPRESSION cells showed lower Rac1 levels compared to LS180 EMPTY cells (Figure 5), whereas MP exposure rescued this effect at 24, 48, and 72 h of treatment (Figures 5D–F, respectively).

**FIGURE 6:**
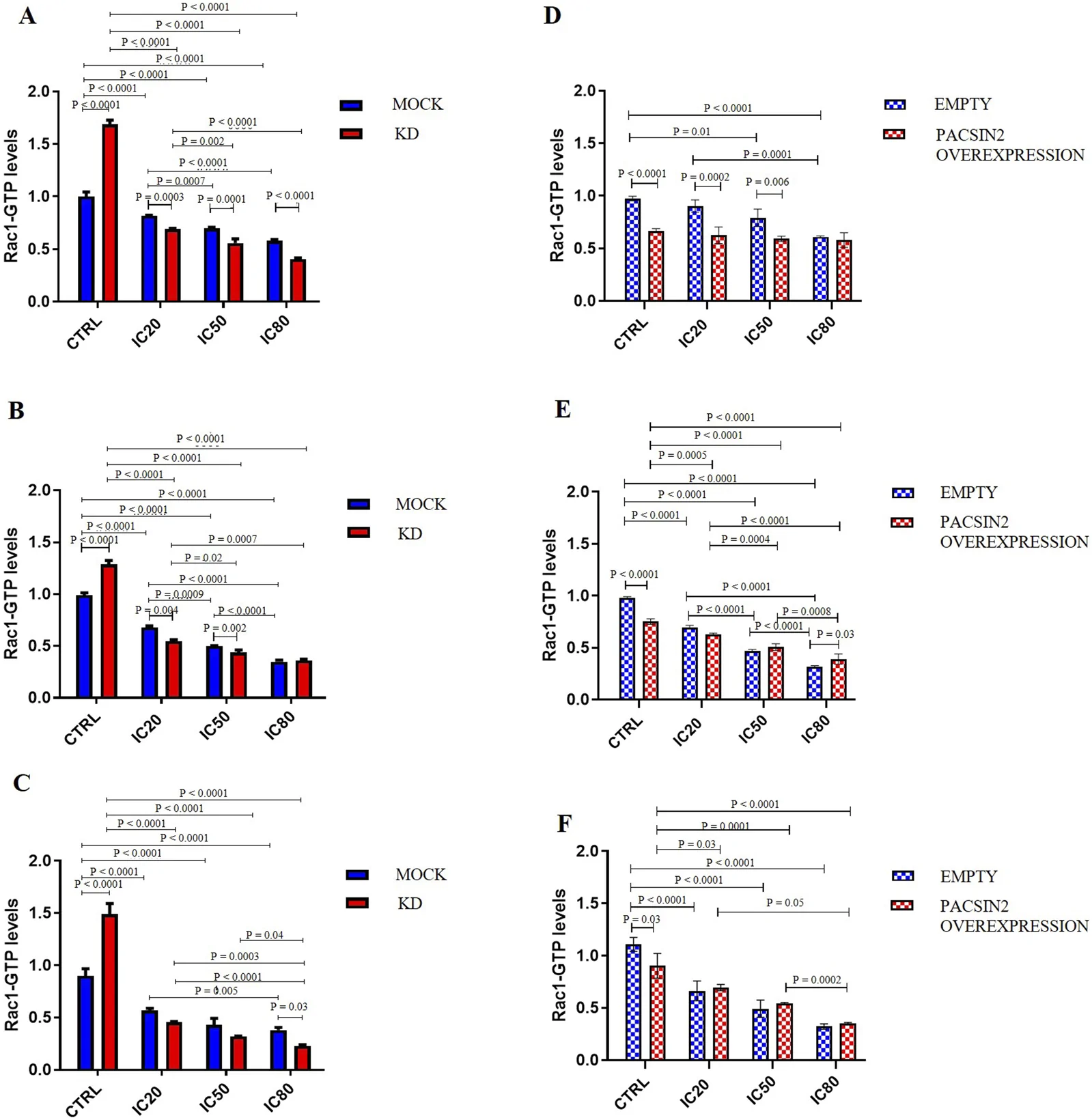
Analysis of Rac1 protein activity after mercaptopurine (MP) treatment. G‐LISA analysis of Rac1 protein activity in LS180 MOCK and LS180 *PACSIN2* KD cell lines untreated and after 24 h **(A)**, 48 h **(B)** or 72 h **(C)** of treatment at IC20, IC50 and IC80 of MP. G‐LISA analysis of Rac1 protein activity in LS180 EMPTY and LS180 PACSIN2 OVEREXPRESSION cell lines untreated and after 24 h **(D)**, 48 h **(E)** or 72 h **(F)** of treatment at IC20, IC50 and IC80 of MP (data expressed as mean ± standard deviation, n =3, Two‐way ANOVA, post‐test Tukey).

Consistent with the protein expression results, Rac1 activity was reduced after MP exposure at all tested concentrations in both LS180 MOCK and KD cell lines after 24, 48, and 72 h of treatment (Figure 6).

Interestingly, after 24 h, there was a difference between LS180 MOCK and KD cell lines under all conditions (Figure 6A); the difference in Rac1 activation was maintained at 24 h in LS180 PACSIN2 OVEREXPRESSION and LS180 EMPTY cells. After both 24 and 48 h, Rac1 active form was lower in LS180 KD cells than in LS180 MOCK cells at IC20 and IC50 (Figures 6A,B) and similar results were found after 72 h of MP exposure at IC80 (Figure 6C). Interestingly, a significant increase in Rac1 activity was detected at 48 h after IC80 exposure in LS180 PACSIN2 OVEREXPRESSION cells (Figure 6E).

### 3.7 Effects of PACSIN2 and MP exposure on cytoskeleton proteins

**FIGURE 7:**
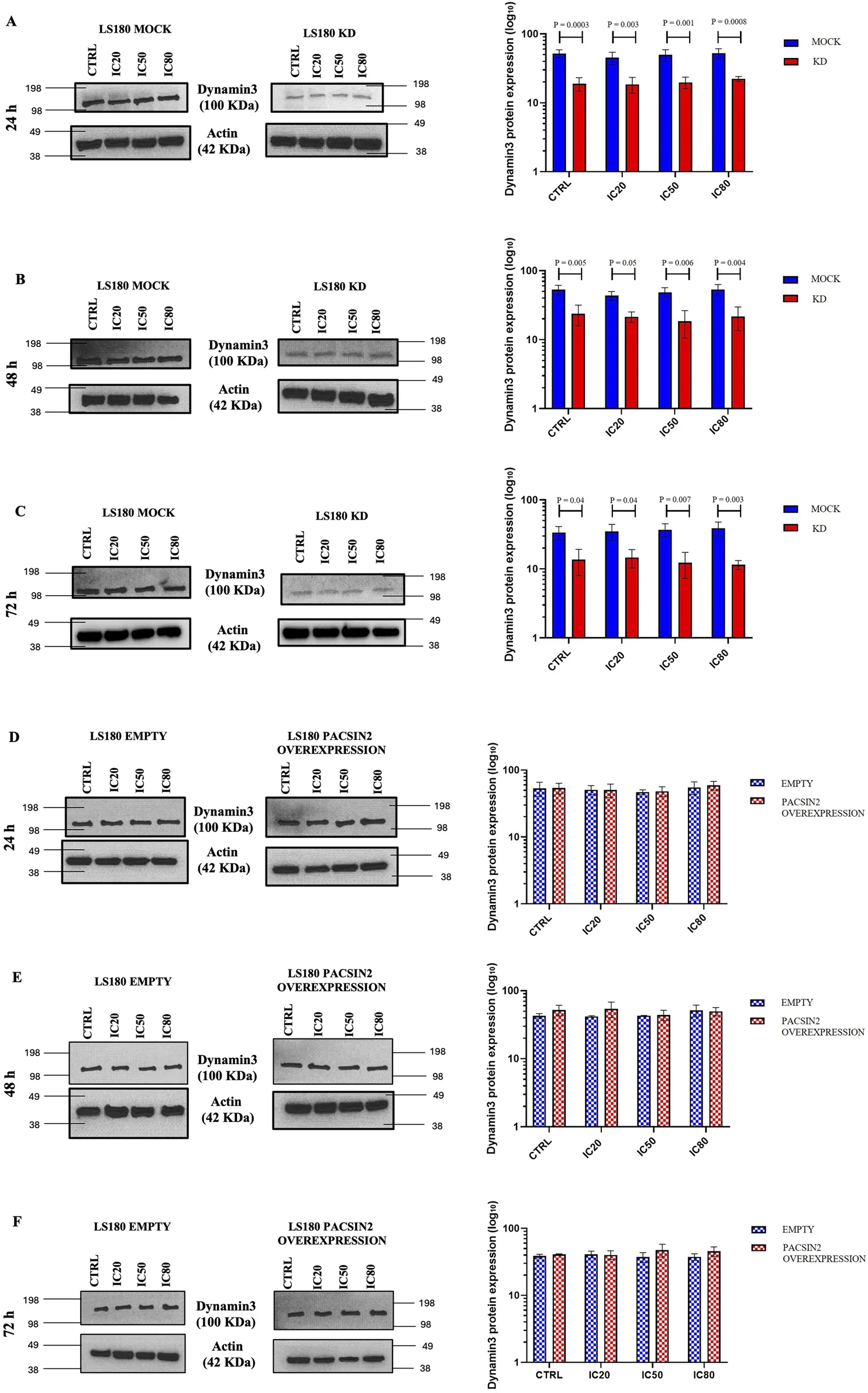
Analysis of Dynamin 3 protein levels under basal condition and after mercaptopurine (MP) treatment. Western Blot analysis of Dynamin 3 protein levels in LS180 MOCK and LS180 *PACSIN2* KD cell lines untreated and after 24 h **(A)**, 48 h **(B)** or 72 h **(C)** of MP treatment at the IC20, IC50 and IC80. Western Blot analysis of Dynamin 3 protein levels in LS180 EMPTY and LS180 PACSIN2 OVEREXPRESSION cell lines untreated and after 24 h **(D)**, 48 h **(E)** or 72 h **(F)** of MP treatment at the IC20, IC50 and IC80. Dynamin 3 levels were normalized using actin and expressed in logarithmic scale (data expressed as mean ± standard deviation, n = 3, Two‐way ANOVA, posttest Tukey). The β‐actin membranes of Panel **(D–F)** are the same presented in Figure 5 Panel **(D–F)**, respectively.

LS180 KD cells showed lower dynamin levels than LS180 MOCK cells (Figure 7), whereas no differences were detected in the dynamin levels between LS180 EMPTY and PACSIN2 OVEREXPRESSION cells (Figure 7). These differences were not affected by MP treatment.

Fluorescence microscopy for tubulin detected using ×20 magnification demonstrated a lower tubulin amount in LS180 KD cells, which was not affected by MP exposure (Supplementary Figure S2). Consistently, LS180 PACSIN2 OVEREXPRESSION cells showed higher tubulin levels that were not altered by MP treatment (Supplementary Figure S2). Moreover, MP exposure at the IC50 for 48 h decreased actin levels in LS180 KD cells (Supplementary Figure S2) and increased the amount in LS180 PACSIN2 OVEREXPRESSION cells (Supplementary Figure S2).

Interestingly, fluorescence microscopy of actin detected using ×50 magnification demonstrated a different localization of this protein in both LS180 KD and LS180 PACSIN2 OVEREXPRESSION cells (Supplementary Figure S2). In particular, the percentage of the cellular area occupied by actin was significantly reduced in LS180 KD cells (Supplementary Figure S2), whereas no significant difference was found in LS180 PACSIN2 OVEREXPRESSION cells (Supplementary Figure S3).

### 3.8 Effects of MP on biomechanical properties

**FIGURE 8:**
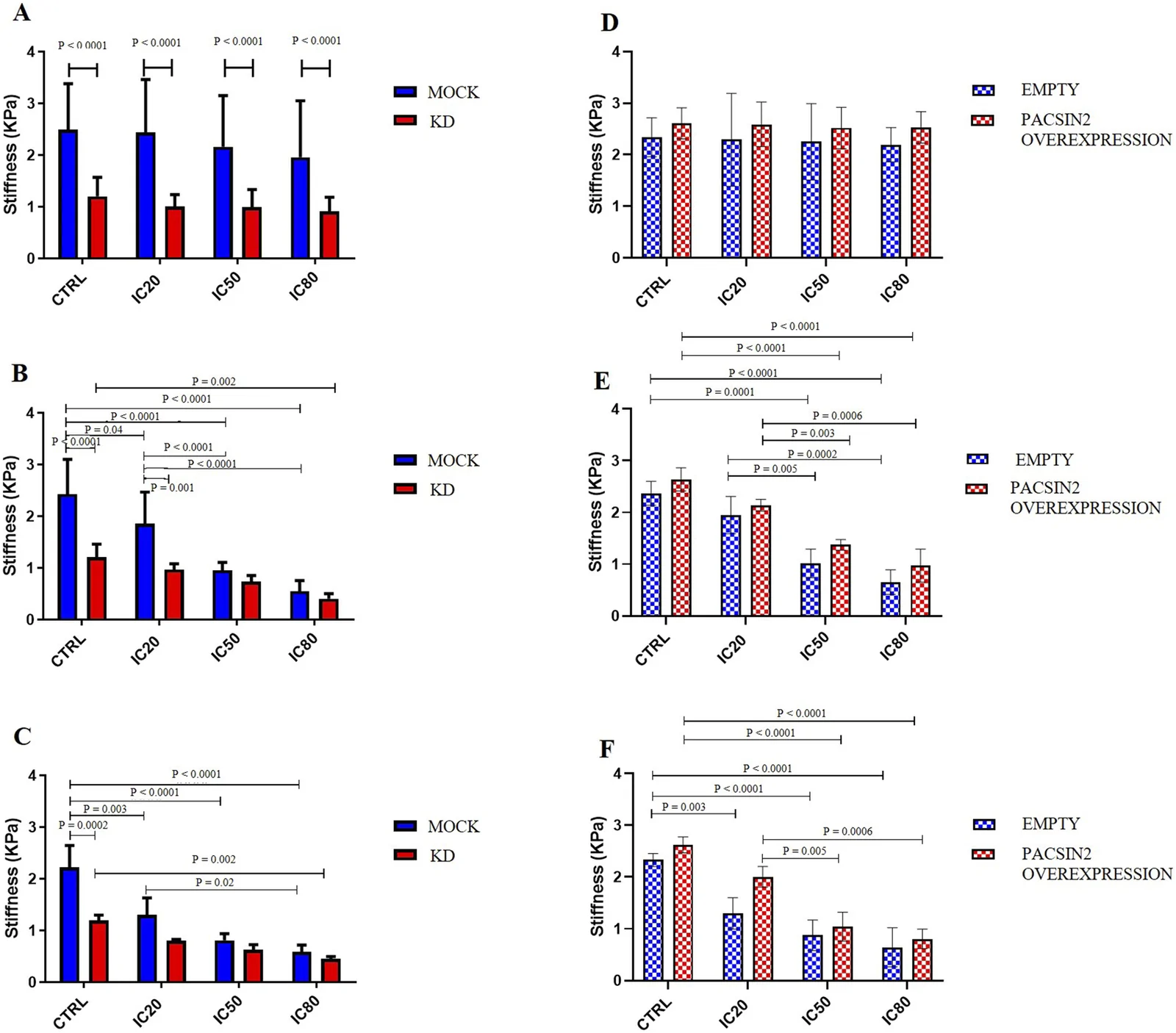
Biomechanical analysis of cell stiffness under basal condition and after mercaptopurine (MP) treatment. Atomic force microscopy (AFM) biomechanical analysis of cell stiffness in LS180 MOCK and LS180 *PACSIN2* KD cell lines untreated and after 24 h **(A)**, 48 h **(B)** or 72 h **(C)** of treatment at IC20, IC50 and IC80 of MP. AFM analyses of cell stiffness in LS180 EMPTY and LS180 PACSIN2 OVEREXPRESSION cell lines untreated and after 24 h **(D)**, 48 h **(E)** or 72 h **(F)** of treatment at IC20, IC50 and IC80 of MP. Data expressed as mean ± standard deviation, n = 3, Two‐way ANOVA, post‐test Tukey.

After 24 h, MP treatment did not affect cellular stiffness in tested cell lines (Figure 8A).

After 48 h and 72 h of treatment, a dose-dependent decrease in stiffness was observed in the LS180 MOCK cell line, whereas for LS180 KD cells a significant reduction in cellular stiffness was observed after MP exposure at IC80 (Figures 8B,C). In addition, after 48 h of drug exposure, the stiffness of LS180 MOCK cells was significantly higher compared to LS180 KD cells after treatment with MP at IC20 (Figure 8B).

LS180 PACSIN2 OVEREXPRESSION cells and LS180 EMPTY cells did not show significant differences in cellular stiffness under basal condition (Figures 8D–F, respectively), whereas MP exposure significantly reduced cell stiffness at 48 and 72 h (Figures 8E,F, respectively) in LS180 PACSIN2 OVEREXPRESSION and LS180 EMPTY cells.

## 4 Discussion

PACSIN2 is a membrane protein involved in cytoskeletal regulation, vesicular trafficking, and autophagy (Zudeh et al., 2023). Previous studies have identified PACSIN2 (Zudeh et al., 2021) and its rs2413739 polymorphism as potential biomarkers of hematological (Smid et al., 2016) and gastrointestinal toxicity in pediatric ALL patients (Franca et al., 2020), as well as of AZA response in pediatric IBD patients (Franca et al., 2019). PACSIN2 KD was shown to increase MP cytotoxicity selectively in intestinal LS180 cells but not in immortalized lymphocytes (Zudeh et al., 2023), suggesting a tissue-specific intestinal role. In the current study, it is reported that PACSIN2 overexpression reverses this phenotype, confirming PACSIN2 as key mediator of thiopurine action in the intestine. Since thiopurines require metabolic conversion to active TGN metabolites (Pelin et al., 2017), MP did not reduce cell viability below 50% after 24 or 48 h; accordingly IC20, IC50, and IC80 values derived from 72h assays were used as reference concentrations for cytotoxic stress. Enhanced MP cytotoxicity in LS180 KD cells was associated with increased cleaved PARP1 levels, suggesting apoptosis induction through the PARP1-mediated pathway (Zudeh et al., 2023). However, the molecular mechanisms by which PACSIN2 modulates thiopurine cytotoxicity are not yet fully understood but likely involve Rac1, a Rho-GTPase negatively regulated by PACSIN2 (de Kreuk et al., 2011). Therefore, the current study investigates the impact of PACSIN2 on Rac1 modulation during MP exposure and its consequences on the biomechanical properties of intestinal cells. The inverse correlation between PACSIN2 and Rac1 governs cytoskeletal regulation and cellular stiffness (de Kreuk et al., 2011), with PACSIN2 negatively modulating Rac1 activity and thereby affecting cell stiffness, adhesion, and migration (Seinen et al., 2016; Kunschmann et al., 2019). Consistent with findings in fibroblasts, T lymphocytes, and cervical and breast cancer epithelial cells (de Kreuk et al., 2011), in the current study, we identified an inverse correlation between PACSIN2 levels and Rac1 expression and activity in intestinal LS180 cells. Accordingly, PACSIN2 overexpression, by reducing Rac1 levels and activity, conferred increased MP resistance, consistent with Rac1 being a key thiopurine target capable of inducing apoptosis in intestinal epithelial cells (Shin et al., 2016). These findings align with the previous evidence that elevated Rac1 levels increase thiopurine sensitivity, whereas Rac1 inhibition enhances cytotoxicity and apoptosis in epithelial cells (Jin et al., 2006). In this study, MP treatment induced a concentration- and time-dependent decrease in Rac1 expression and activity in both KD and MOCK cells, with more pronounced effects in KD cells, suggesting that these cells are particularly dependent on Rac1 signalling for survival. The additive cytotoxic effect observed between MP and the Rac1 inhibitor NSC23766 further supports this interpretation. Interestingly, PACSIN2 OVEREXPRESSION reduced basal Rac1 expression and activity levels, which were further decreased upon MP exposure. Collectively, these findings suggest that patients carrying PACSIN2 risk genotypes (CT or TT), who putatively express lower PACSIN2 and higher Rac1 levels, might benefit from alternative immunosuppressants that do not target the Rac1 pathway.

Taken together, this evidence indicates that PACSIN2 silencing increased Rac1 levels, thereby increasing thiopurine cytotoxicity in intestinal LS180 cells and supporting the intestine-specific role of PACSIN2 in thiopurine response (Zudeh et al., 2023).

At the molecular level, the SH3 domain of PACSIN2 interacts with the C-terminal domain of Rac1, regulating both its expression and GTPase activity (de Kreuk et al., 2011). The higher Rac1 levels in LS180 KD cells likely underlie their increased thiopurine sensitivity: the thiopurine metabolite 6-thioguanine triphosphate (6-TGTP) forms a disulphide adduct with the redox-sensitive GXXXXGK(S/T)C motif of Rac1, blocking GTPase activity and inducing apoptosis, as shown in CD3/CD28-stimulated T-cells (Shin et al., 2016; Tiede et al., 2003). Although RhoA and Cdc42 share this motif, Rac1 is the predominant active Rho GTPase in stimulated T-cells and in the intestinal epithelium (Shin et al., 2016; Ray et al., 2003) supporting its role as the primary thiopurine target (Jin et al., 2006). However, the relative contribution of RhoA and Cdc42 to thiopurine cytotoxicity in intestinal models warrants further investigation, as does the direct measurement of Rac1 activity using purified GTPases in the presence or absence of MP.

AFM measurements revealed that LS180 MOCK cells were stiffer than KD cells, probably because PACSIN2 depletion destabilizes microtubules and reduces actin filament stability and polymerization, consistent with findings in murine intestinal epithelial cells, where PACSIN2 KD impaired cytoskeletal network formation (Postema et al., 2019). PACSIN2 overexpression conversely tended to increase cell stiffness. To capture biomechanical changes preceding MP-induced cytotoxicity, stiffness was assessed at 24 and 48 h, earlier than the 72h time point at which cytotoxic effects became apparent. Several drugs reduce cell stiffness by disrupting actin filaments and cytoskeletal organization, decreasing the average elastic modulus (Bonsergent et al., 2021; Kubiak et al., 2020; Poppe et al., 2006; Mohammadi et al., 2021). Accordingly, in this study, MP induced a dose- and time-dependent reduction in cell stiffness in both LS180 MOCK and KD cell lines at 48 and 72 h, with more pronounced effects in LS180 KD cells. No significant changes were observed after 24 h of MP treatment, probably because this interval is not sufficient for cytoskeletal reorganization to manifest structurally, despite rapid Rac1 inhibition at this time point (Leyden et al., 2021). This temporal pattern is consistent with the indirect mechanism of MP: unlike cytoskeleton-targeting agents, such as vincristine or doxorubicin, which reduce stiffness within 12 h (Alhalhooly et al., 2021), MP acts by modulating Rho GTPase signalling, including Rac1, Rho, and Cdc42, resulting in delayed biomechanical effects (Li et al., 2016; Shurin et al., 2008).

To understand the mechanism by which PACSIN2 and MP influence cell stiffness, key cytoskeletal proteins, particularly dynamin, tubulin, and actin, were investigated. We found reduced dynamin levels in LS180 KD cells under basal conditions, as previously demonstrated by Postema et al. on murine cells with PACSIN2 knock-out (Postema et al., 2019). However, dynamin levels were unaffected by PACSIN2 OVEREXPRESSION or MP treatment, suggesting that dynamin is not the primary effector of PACSIN2- or MP-mediated stiffness changes. In the current study, PACSIN2 KD was also associated with reduced tubulin levels, consistent with evidence that PACSIN2 silencing impairs tubulin polymerization in NIH 3T3 fibroblasts (Grimm-Günter et al., 2008), whereas MP alone had no significant effect on tubulin abundance. In contrast, actin levels were sensitive to both PACSIN2 modulation and MP treatment, identifying actin as the primary determinant of the observed changes in cellular stiffness. Rac1 is a key regulator of actin polymerization and cytoskeletal organization; its downregulation promotes actin stress fiber formation and redistribution (Lawson and Ridley, 2018; Ridley, 2015), a pattern consistent with the elevated basal Rac1 levels observed in PACSIN2 KD cells. In these cells, the absence of PACSIN2-mediated negative regulation leads to Rac1 hyperactivation and accumulation of Rac1-GTP at the plasma membrane, sustaining downstream actin remodelling (Castro-Castro et al., 2016). In parallel, PACSIN2 also directly regulates actin filaments stability through its F‐BAR domain (Kostan et al., 2014); consequently, its absence results in reduced actin filament stability, a softer cytoskeleton, and decreased cellular rigidity (Kostan et al., 2014), as observed in LS180 KD cells. The finding that KD cells display increased Rac1 activity yet reduced stiffness suggests that PACSIN2-mediated actin stabilization exerts a greater influence on cell mechanics than Rac1 signalling alone. This interpretation is further supported by the observation that MP-induced Rac1 inhibition, conventionally expected to increase stiffness, did not restore stiffness in KD cells, underscoring the dominant role of PACSIN2 in governing cellular mechanics in this intestinal model. This study also places MP-induced biomechanical alterations in a broader pharmacological context, suggesting that changes in cell stiffness may represent an integrative indicator of cumulative cytoskeletal stress induced by drugs that act indirectly on the cytoskeleton.

Several limitations should be acknowledged. Although LS180 cells are a tumor-derived model, they are widely recognized as a valuable model for studying intestinal drug metabolism and assessing drug-induced cytotoxicity (van de Kerkhof et al., 2007); future validation analyses should be performed in intestinal organoids, which better recapitulate the intestinal epithelium physiology.

AFM measurements were performed on 4%-PFA-fixed cells. However, PFA may elevate absolute stiffness values, indicated as Young modulus, by cross-linking cell surface proteins and immobilizing F-actin filaments in a PFA concentration depended way (Kim et al., 2017). Nevertheless, since all conditions were processed identically, relative comparisons remain reliable. The inclusion of specific cytoskeletal inhibitors, such as Cytochalasin D, in future experiments would help to discriminate the intracellular origin of stiffness changes, complementing the AFM presented data (Senigagliesi et al., 2022; Apollonio et al., 2023; Musto et al., 2021). The cytoskeleton organization was evaluated by analysing the cytoskeletal filament (such as actin) distribution, which was obtained by calculating the fiber percentage in the cellular area. This approach allowed us to define how cytoskeletal fiber organization changes across conditions (Gunasekara et al., 2024; Witko et al., 2020). However, further analyses with more sophisticated methods should be performed, taking care that LS180 cells show a large nuclear compartment and limited cytosolic area, constraining the resolution of cytoskeletal distribution analyses. Finally, investigating the subcellular localization of Rac1 and PACSIN2-interacting proteins, such as filamin A would clarify the spatial coordination of these proteins during MP response and their contribution to biomechanical changes.

## 5 Conclusion

This study shows that PACSIN2 modulates MP response in intestinal cells by regulating Rac1 expression and activity, and by affecting cytoskeletal organization through actin stabilization. PACSIN2 KD increased Rac1 levels and activity, enhancing thiopurine-mediated apoptosis and MP cytotoxicity, while actin emerged as the primary cytoskeletal determinant of the observed changes in cell stiffness. These findings provide a molecular framework linking PACSIN2 expression, Rac1 signalling and cytoskeletal mechanics to thiopurine sensitivity in the intestinal epithelium, supporting PACSIN2 as a potential biomarker of thiopurine-induced gastrointestinal toxicity and a target for personalized therapeutic strategies.

## Data Availability

The raw data supporting the conclusions of this article will be made available by the authors, without undue reservation.
