# Supplementary material for: PACSIN2 regulates mercaptopurine cytotoxicity and cellular biomechanics through Rac1 modulation in intestinal epithelial cells
Source: Front Pharmacol. 2026 Sep 1;17:1860702. doi: 10.3389/fphar.2026.1860702 (PMC13575966; doi:10.3389/fphar.2026.1860702)
Supplement: Supplementary file 1 [file Supplementaryfile1.pdf]

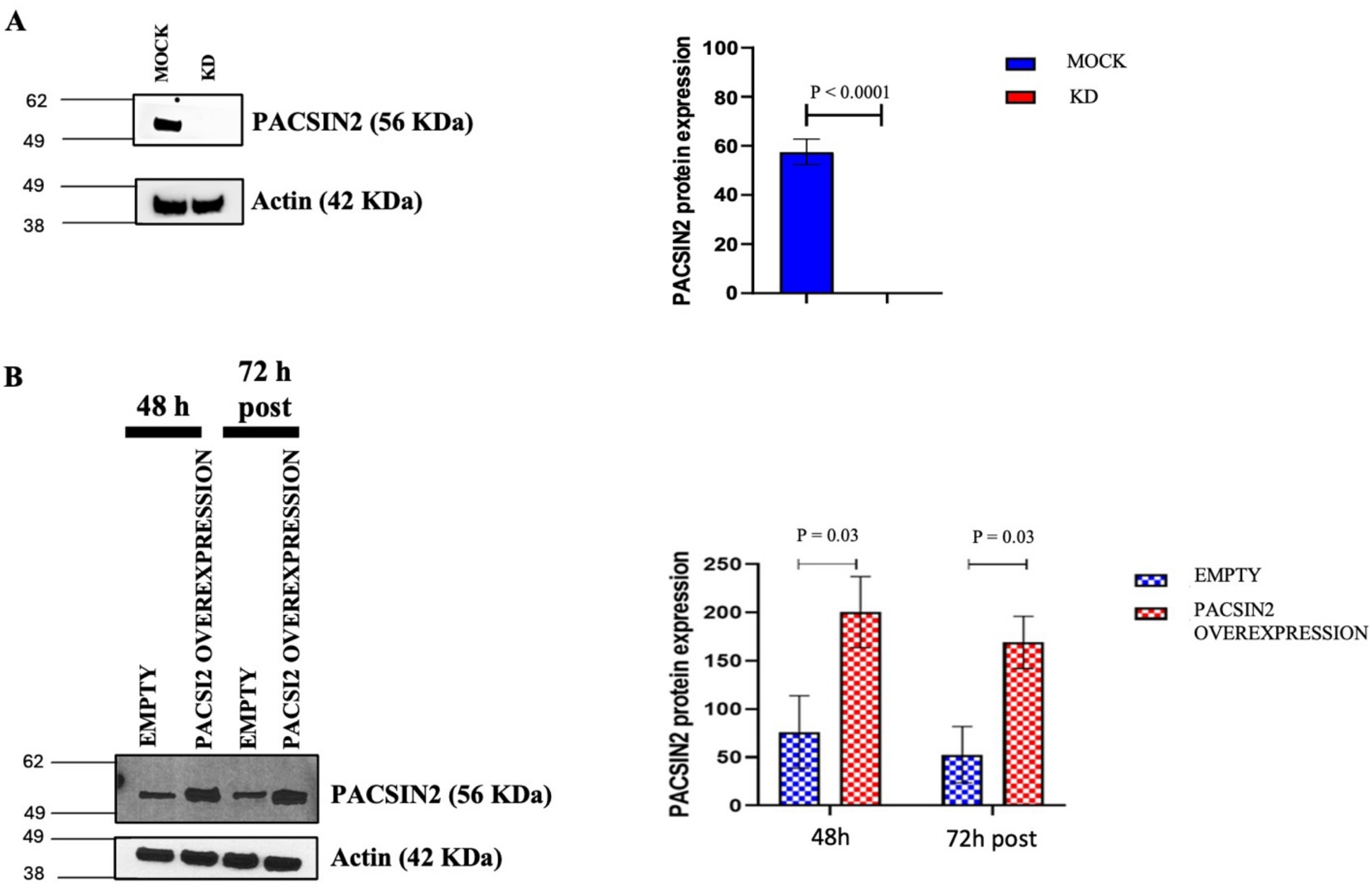

Supplementary Fig. 1. Cell line characterization. (A) The stable *PACSIN2* KD, obtained by transfecting cells with PACSIN2-specific shRNAs, was confirmed evaluating PACSIN2 levels by western blot using 10 µg of cell lysate for LS180 cells. In control cells, scramble shRNAs were used (MOCK). Actin was used as housekeeping. **(B)** LS180 MOCK cells were transfected with an empty plasmid (LS180 EMPTY cells) as control or with a plasmid encoding for PACSIN2 wild-type (LS180 PACSIN2 OVEREXPRESSING cells). The transient overexpression of PACSIN2 was confirmed evaluating PACSIN2 protein levels at 48 hours after transfection (48 h) and after additional 72 hours (72h post). Ten µg of cell lysate was used and bands were normalized against β-actin (data expressed as mean ± standard deviation, n = 3, t-test, 48 h, 72 h post).

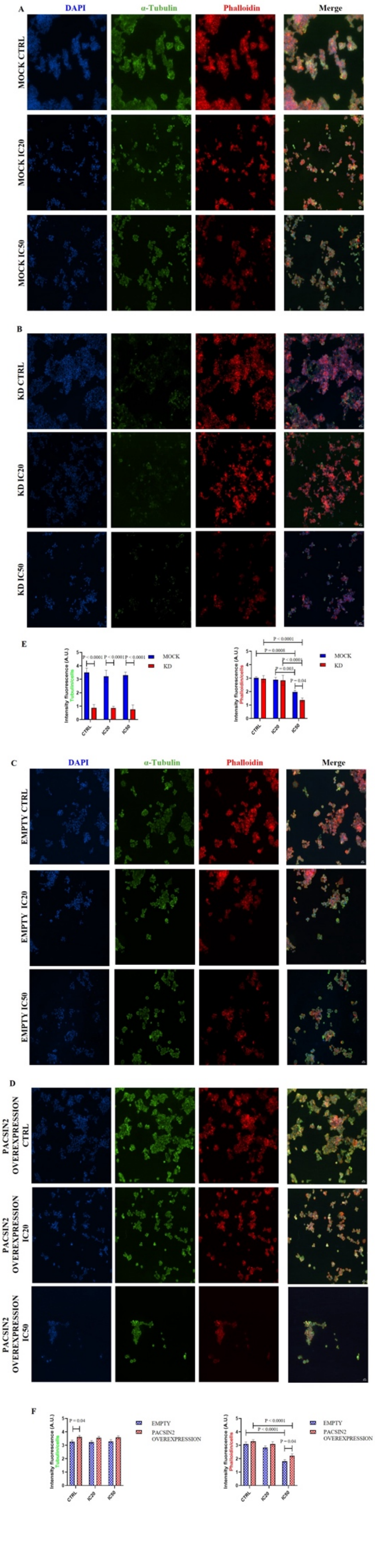
A
DAPI
α-Tubulin
Phalloidin
Merge
MOCK CTRL
MOCK IC20
MOCK IC50
B
KD CTRL
KD IC20
KD IC50
E
MOCK
KD
CTRL
IC20
IC50
C
EMPTY CTRL
EMPTY IC20
EMPTY IC50
D
PACSIN2 OVEREXPRESSION CTRL
PACSIN2 OVEREXPRESSION IC20
PACSIN2 OVEREXPRESSION IC50
F
EMPTY
PACSIN2 OVEREXPRESSION

Supplementary Fig. 2. Fluorescence microscopy analyses of tubulin and actin under basal condition and after mercaptopurine (MP) treatment. (A) Intensity immunofluorescence analysis of tubulin and actin protein levels in LS180 MOCK and LS180 *PACSIN2* KD cell lines untreated and after 48 hours of MP treatment at the IC20 and IC50. **(B)** Intensity immunofluorescence analysis of tubulin and actin protein levels in LS180 EMPTY and LS180 PACSIN2 OVEREXPRESSING cell lines untreated and after 48 hours of MP treatment at the IC20 and IC50. The detection was obtained using 20X magnification and 100 milliseconds exposure. Fluorescence intensity of every protein was normalized against the cell number/field (data expressed as mean ± standard deviation, n = 3, Two-way ANOVA, post-test Tukey).

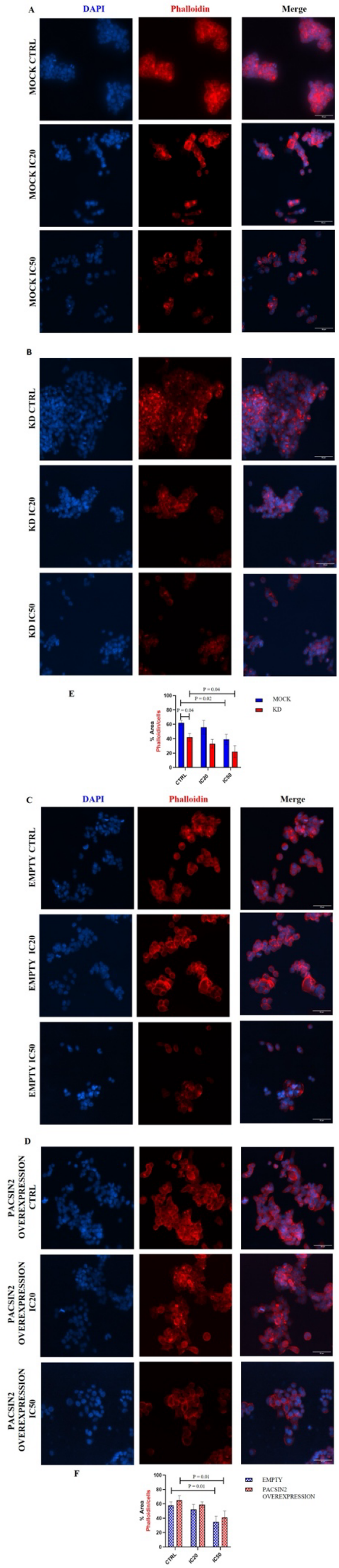

A
DAPI
Phalloidin
Merge
MOCK CTRL
MOCK IC20
MOCK IC50
B
KD CTRL
KD IC20
KD IC50
E
P = 0.04
P = 0.02
P = 0.04
MOCK
KD
% Area
CTRL
IC20
IC50
C
DAPI
Phalloidin
Merge
EMPTY CTRL
EMPTY IC20
EMPTY IC50
D
PACSIN2 OVEREXPRESSION CTRL
PACSIN2 OVEREXPRESSION IC20
PACSIN2 OVEREXPRESSION IC50
F
P = 0.01
P = 0.01
EMPTY
PACSIN2 OVEREXPRESSION
% Area
CTRL
IC20
IC50

Supplementary Fig. 3. Fluorescence microscopy analyses of actin cellular distribution under basal condition and after mercaptopurine (MP) treatment. (A) Immunofluorescence analysis of actin protein cellular distribution in LS180 MOCK and LS180 *PACSIN2* KD cell lines under basal condition and after 48 hours of MP treatment at the IC20 and IC50. **(B)** Immunofluorescence analysis of actin protein cellular distribution in LS180 EMPTY and LS180 PACSIN2 OVEREXPRESSING cell lines under basal condition and after 48 hours of MP treatment at the IC20 and IC50. The detection was obtained using 50X magnification and 100 milliseconds exposure. The percentage of cellular area occupied by actin was normalized against the cell surface area (data expressed as mean ± standard deviation, n = 3, Two-way ANOVA, post-test Tukey).

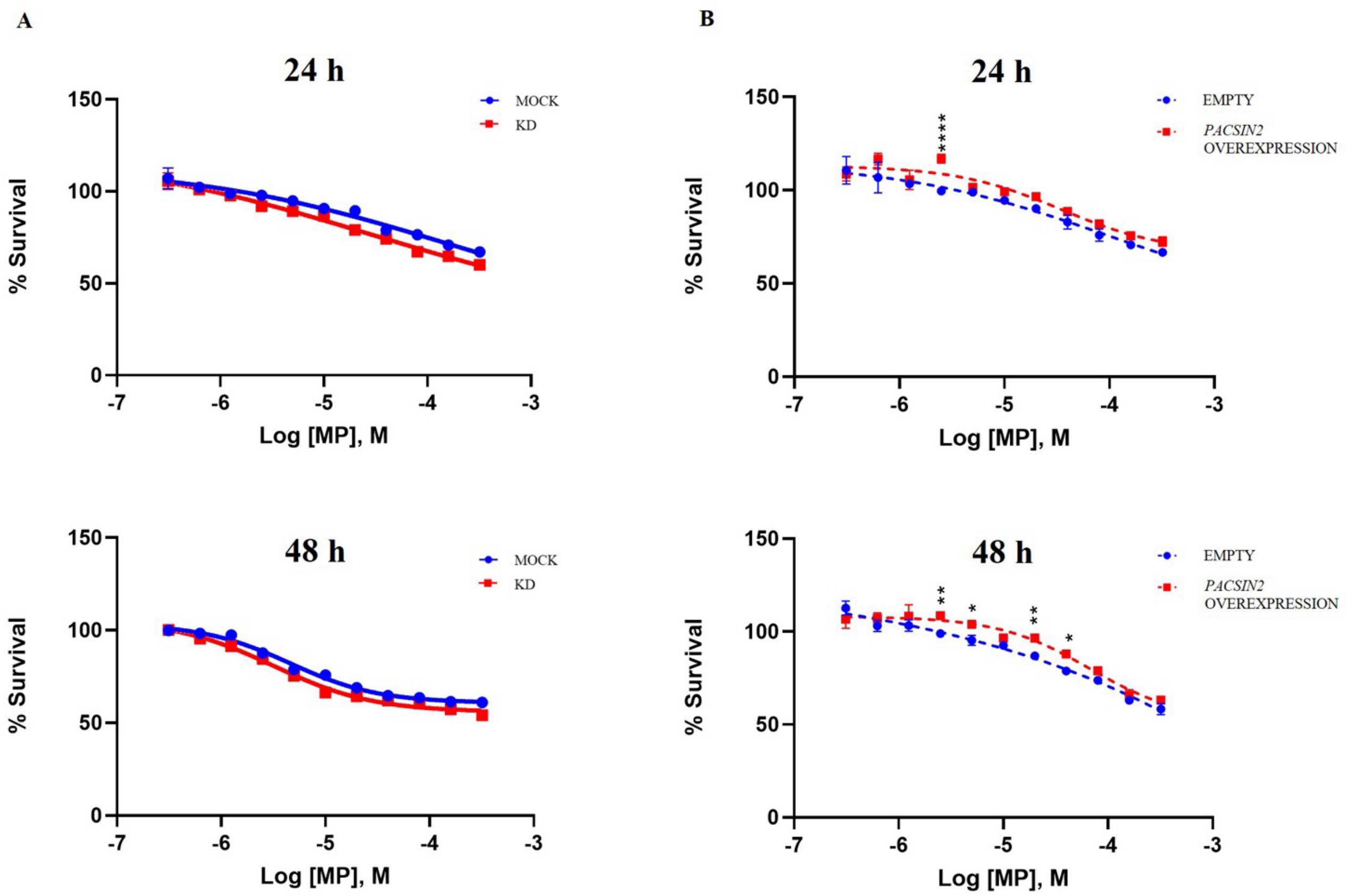

Supplementary Fig. 4. The effect of PACSIN2 on mercaptopurine (MP) cytotoxicity after 24 hours and 48 hours of drug exposure. (A) Cytotoxicity MTT dose-response curve of LS180 MOCK and LS180 PACSIN2 KD cells, cells treated with MP (from 0.3 µM to 160 µM) for 24 hours or 48 hours (Two-way ANOVA, post-test Tukey). **(B)** Cytotoxicity MTT dose-response curve of LS180 MOCK EMPTY and LS180 MOCK PACSIN2 OVEREXPRESSION cells treated with MP (from 0.3 µM to 160 µM) for 24 hours or 48 hours (Two-way ANOVA, post-test Tukey).
