# Supplementary material for: PACSIN2 regulates mercaptopurine cytotoxicity and cellular biomechanics through Rac1 modulation in intestinal epithelial cells
Source: Front Pharmacol. 2026 Sep 1;17:1860702. doi: 10.3389/fphar.2026.1860702 (PMC13575966; doi:10.3389/fphar.2026.1860702)
Supplement: Supplementary file 2 [file Table1.docx]

**Supplementary Table**

| **24h of drug exposure** | | | | | | | |
| --- | --- | --- | --- | --- | --- | --- | --- |
|  | **Concentration of mercaptopurine (μM)**  **LS180 MOCK** | | **Concentration of mercaptopurine (μM)**  **LS180 KD** | | **Concentration of mercaptopurine (μM)**  **LS180 MOCK EMPTY** | | **Concentration of mercaptopurine (μM)**  **LS180 PACSIN2 OVEREXPRESSION** |
| **IC20** | 89 | | 20.2 | | 38.2 | | 29.5 |
| **IC50** | 159.8 | | 29.34 | | 98.53 | | 34.6 |
| **IC80** | 287.3 | | 42.2 | | 255 | | 172.9 |
| **48h of drug exposure** | | | | | | | |
| **IC20** | | 2.7 | | 2.0 | | 2.9 | 3.6 |
| **IC50** | | 4.6 | | 3.2 | | 4.6 | 6.7 |
| **IC80** | | 5.9 | | 5.2 | | 7.04 | 16 |

| **72h of drug exposure** | | | | |
| --- | --- | --- | --- | --- |
| **IC20** | 2.27 | 1.28 | 2.7 | 3.1 |
| **IC50** | 3.23 | 2.18 | 4 | 7.8 |
| **IC80** | 5.66 | 4.90 | 5.8 | 10.6 |

**Supplementary Table 1.** **Concentrations of mercaptopurine (MP) for which cell viability was reduced by 20­50­80% respectively in LS180 cell lines.** Concentrations of MP reducing cell viability by 20% (IC20) 50% (IC50) and 80% (IC80) compared to the untreated control in LS180 MOCK, LS180 KD, LS180 MOCK EMPTY and LS180 MOCK PACSIN2 cell lines after 24, 48 and 72 hours of drug exposure.
